# Transitory Spontaneous Remission of Myelodysplasia in an Elderly Man while Receiving Intravesical Bacillus Calmette-Guérin for Bladder Cancer: A Case Report and Review of the Literature

**DOI:** 10.1155/2018/9750532

**Published:** 2018-11-11

**Authors:** Nigel P. Murray, Cynthia Fuentealba, Isidora Salazar, Aníbal Salazar, Marco Antonio Lopez, Simona Minzer

**Affiliations:** ^1^Consultant Hemologist, Depto. Medicine, Hospital de Carabineros de Chile, Simón Bolívar 2200, Ñuñoa 7770199, Santiago, Chile; ^2^Professor Hematology, Faculty of Medicine, University Finis Terrae, Av Pedro de Valdivia 1509, Providencia, Santiago, Chile; ^3^Consultant Urologist, Urology Service, Hospital de Carabineros de Chile, Simón Bolívar 2200, Ñuñoa 7770199, Santiago, Chile; ^4^Medical Student, Faculty Medicine, University Los Andes, Monseñor Alvaro del Portillo 12,455 Las Condes, Santiago, Chile; ^5^Physician, General Medicine, Hospital de Carabineros de Chile, Simón Bolívar 2200, Ñuñoa 7770199, Santiago, Chile

## Abstract

Myelodysplasia is a clonal disorder characterized by progressive cytopenias. Intravescial BCG is standard immunotherapy for superficial bladder cancer. We present a patient with transfusion-dependent myelodysplasia whose blood counts normalized during treatment with intravesical BCG for bladder cancer. After finishing treatment, the patient became transfusion dependent once more. We discuss possible mechanisms to explain this case report.

## 1. Introduction

Intravesical treatment with Bacillus Calmette-Guérin (BCG) has been a standard adjuvant therapy for non-muscle-invasive bladder cancer for the last 40–50 years [1]. BCG is a live attenuated strain of *Mycobacterium bovis*, and although minor side effects are common, serious side effects occur in less than 5% of patients, of which most are local and present early after treatment [2]. Systemic side effects have included infection, autoimmune disorders, and pancytopenia [3, 4].

Although spontaneous remission of acute myeloid leukemia was first reported in 1878 [5], it is a rare event and usually of short duration, of the approximately 100 cases reported before the use of chemotherapy in the 1950s; only 14 eligible cases fulfilling modern criteria have been reported up until 2014 [6]. Fewer cases have been reported in patients with myelodysplasia, the majority in children or young people <20 years and in the presence of monosomy 7 [7–9]. In a large retrospective series of 564 adult patients with myelodysplasia, spontaneous remission was reported in 9 (1.6%) of cases [10]. We report the case of a 69-year-old man with myelodyplasia whose full blood count became normal during treatment with intravesical BCG for bladder cancer.

## 2. Clinical Case

A 69-year-old man presented in 2008 with a macrocytic anemia; the hemoglobin level was 10.2 g/dl (13.0–18.0 gr/dl), MCV (mean corpuscular volume) 114 fl (80–97 fl), the white cell count including differential count was normal, the platelet count was 155,000 (normal), and the reticulocyte count was decreased (0.7%) in the presence of an anemia. The serum B12, serum folate, serum thyroid-stimulating hormone level, and liver function tests were normal. A bone marrow biopsy was consistent with refractory anemia and blasts <5%. He was treated with a trial of anabolic steroids without success.

**Figure 1:**
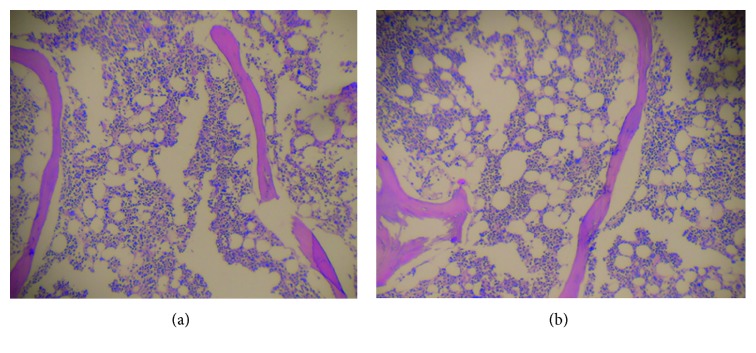
First bone marrow biopsy showing normo/hypercellularity.

In 2011, the patient was referred to the hematology department, with a hemoglobin level of 7.0 gr/dl, MCV 123 fl, a platelet count of 50,000, and reticulocyte count of 0.9%. At this time, serum B12, serum folate, serum TSH, and liver function tests were normal. The ferritin was 446 ng/ml (increased), percent saturation of transferrin was 31.8 (normal), a serum protein electrophoresis was normal, urine analysis was normal, and serum PSA level was 0.699 ng/ml (the patient had previously undergone a transurethral resection for benign prostatic hyperplasia in 1996). Repeat bone marrow biopsy, after red cell and platelet transfusions, revealed a hypercellular bone marrow, with dysplastic features, including micromegakaryocytes and blasts <5%. Cytogenetic study revealed a normal karyotype. The diagnosis remained that of myelodysplasia of refractory anemia (Figure 1).

To alleviate the symptoms of anemia, monthly transfusions of red cells were needed to maintain the hemoglobin level above 8.0 gr/dl. The platelet count continued to decrease but apart from some superficial bruising and platelet transfusions were not required.

In October 2013, the patient noted painless macroscopic hematuria lasting for two days, his full blood count showed a hemoglobin level of 7.5 gr/dl; a platelet count of 13,000, a normal white cell count, and tests of the coagulation showed a normal prothrombin and activated partial thromboplastin times.

**Figure 2:**
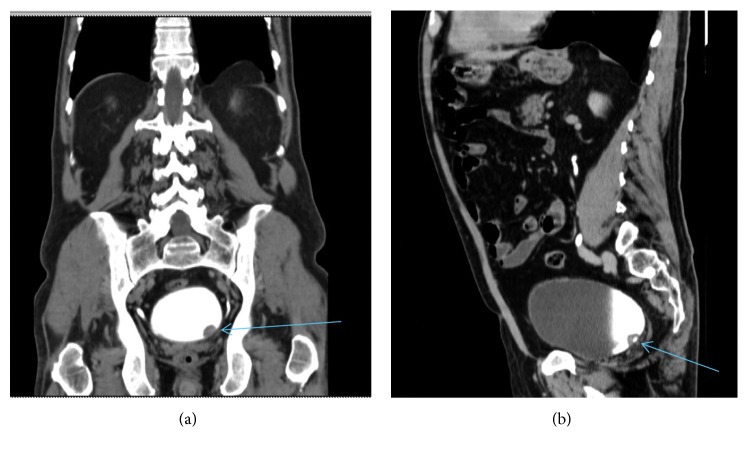
Coronal sections Uro-CT scan with contrast showing filling defect (arrows).

**Figure 3:**
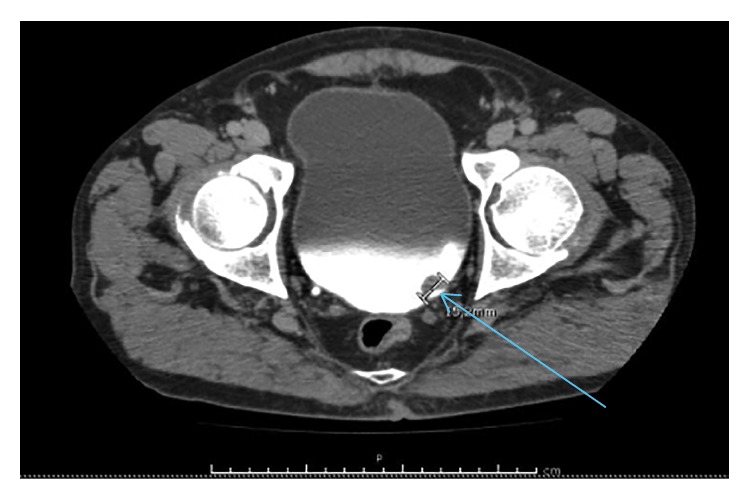
Sagittal section Uro-CT scan showing proliferative lesion left ureteral meatus (arrow).

Urine analysis confirmed hematuria, and urine culture was negative for infection. An excretion CT scan of the urinary system revealed a lobulated lesion in the region of the left ureteral meatus with a diameter of 16 mm (Figures 2 and 3).

**Figure 4:**
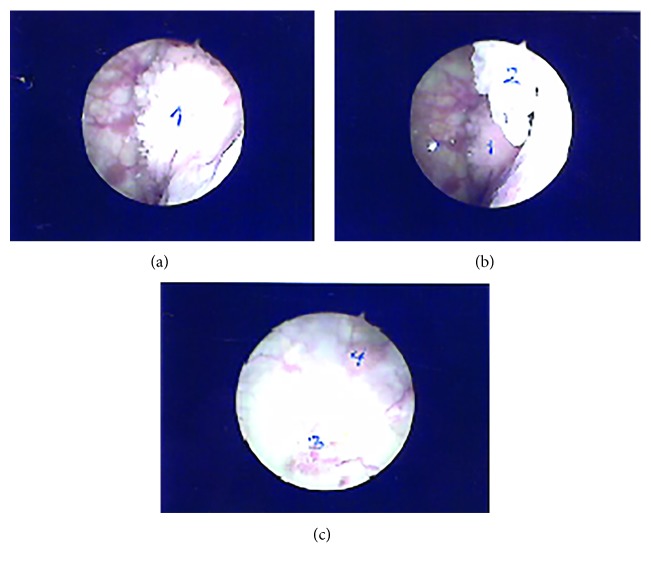
Four tumors seen at initial cystoscopy.

With transfusions of red cells and platelets, the patient underwent a diagnostic cystoscopy, which revealed five bladder tumors (Figure 4).

With transfusional support of both red cells and platelets, a TUR-B (transurethral resection bladder tumor) was performed, with resection of the five tumors and electrocoagulation of the tumor bed. The largest tumor had a diameter of 1 cm and was located at the left urethral opening. Pathological analysis revealed a low-grade superficial urothelial papillary carcinoma without evidence of bladder wall infiltration.

After resection of the tumor, the patients' transfusional requirements decreased, but he remained transfusion dependent. A second cystoscopy two months later showed the tumor resection scar; in the trigonal area, a small papillary growth was resected, and the area around the scar was electrocauterized. The growth was a superficial low-grade papillary carcinoma.

Intravesical BCG, weekly for 6 weeks, then two-weekly for 4 doses, and then monthly for 1 year, starting in March 2015 and finishing in July 2016. Repeat cystoscopy in October 2015 and February 2017 showed no tumor recurrence.

**Figure 5:**
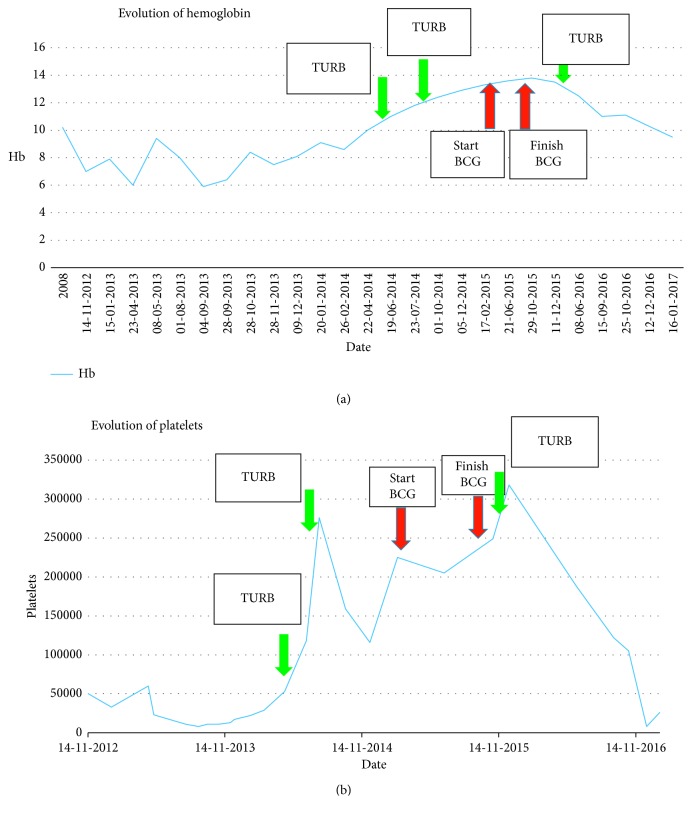
Trends in hemoglobin levels and platelet counts with time.

**Figure 6:**
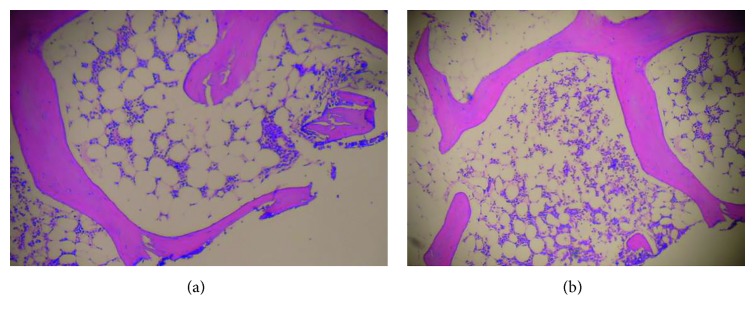
Repeated bone marrow biopsy showing marked decreased cellularity in comparison with the first biopsy.

While receiving BCG, the hemoglobin and platelet counts increased, achieving normal levels and the patient became transfusion independent. One month after completing BCG treatment, both the hemoglobin level and platelet counts were decreasing, and six months later, the patient had a hemoglobin level of 8.5 gr/dl, MCV 113, and a platelet count of 26,000 (Figure 5). A repeat bone marrow biopsy showed a hypocellular bone marrow, with between 30% and 50% of the intertrabecular spaces being occupied by hematopoietic tissue (Figure 6). There was a significant decrease in the erythroid precursors (<10%) and megakaryocytes. There was no evidence of fibrosis or infiltration of the bone marrow with a CD34 count of less than 5%.

**Figure 7:**
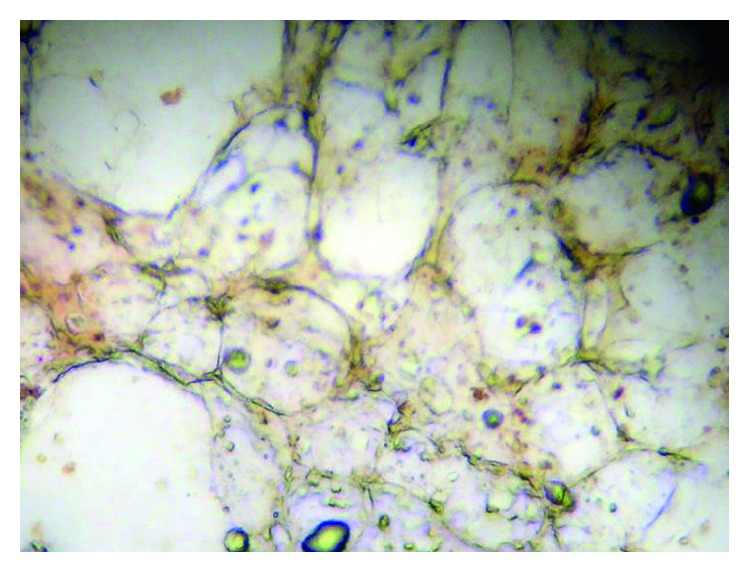
Bone marrow, negative for micrometastasis.

Immunohistochemistry detection of cells staining positive for pancytokeratin and EpCAM was negative, indicating the lack of micrometastatic disease (Figure 7).

Since February 2017, the patient is once again transfusion dependent for packed red cells.

## 3. Discussion

Myelodysplasia is the result of a clonal stem cell defect, which may be accompanied by various cytogenetic abnormalities. It is a progressive disease mainly affecting the elderly, and in the majority of patients, the treatment is transfusional support with red cells and/or platelets to maintain the quality of life.

The clinical findings in this case were consistent with myelodysplasia with progressive anemia, requiring chronic transfusions of red cells to maintain the quality of life. Platelet transfusions were on a required basis according to hemorrhagic events and not on a prophylactic basis. The initial macroscopic hematuria was considered to be in the context of a possible urinary infection and the severe thrombocytopenia, which in myelodysplasia is associated with platelet dysfunction.

The final diagnosis was multiple foci of bladder cancer, which was treated according to the recommended clinical guidelines of resection and immunotherapy with BCG.

The case presents two questions, Why did transfusion requirements decrease after tumor resection and were unnecessary during BCG treatment? Secondly, why did the hemoglobin and platelet counts return to baseline levels after finishing BCG treatment and why was there a change in the cellularity in the bone marrow?

1. Decreased transfusional requirements after tumor resection: a possible reason to explain the recuperation of the red cells and platelets after tumor resection is that, by removing the tumor, there was no further bleeding (including microscopic). The decreased consumption of platelets and decreased loss of red cells resulted in increased levels of both blood components. The limited capacity of the myelodysplastic bone marrow to increase production due to bleeding resulted in a more severe anemia and thrombocytopenia.

However, this theory falls short because it does not explain the rapid decrease in red cell and platelet levels after BCG therapy, in the light of tumor remission and without evidence of bone marrow infiltration by tumor cells. Nor does it explain the change from a hypercellular to a hypocellular bone marrow.

Bladder cancer has been associated with a paraneoplastic leukemoid reaction, there being a leukocytosis [11, 12]. Bladder cancer has also been associated with thrombocytosis, alone [13] or in combination with the leukemoid reaction [14]. The leukocytosis is thought to be due to production of granulocyte colony stimulating factor by the tumor [15, 16]. However, in the case reported, the platelet count increased after tumor resection.

(2) Effect of intravesical BCG therapy: with the use of BCG, the hemoglobin and platelet counts increased to normal levels, the maximum platelet count being 317,000, and maximum hemoglobin level of 13.7 gr/dl (although the MCV remained high). These levels were maintained throughout the year-long period, decreasing to transfusion-dependent levels within 3 months of finishing treatment.

The use of intravescial BCG has been reported to be associated with systemic autoimmune diseases, such as cyroglobulinemia vasculitis [17], autoimmune retinopathy [18], Henoch-Schonlein purpura [19], and haemophagocytic syndrome [20]. BCG has also caused a pancytopenia due to bone marrow infection, which may be delayed up to two years post-treatment [4, 20]. This highlights that intravesical BCG may produce systemic side-effects. However, these mechanisms do not explain the normalization of the full blood count and zero transfusional requirements during BCG therapy and subsequent worsening of the anemia and thrombocytopenia after finishing the treatment.

The mechanisms for spontaneous remissions in acute myeloid leukemia are unknown; however, severe systemic infections often precede the remission. They are most often reported with bacterial infections [21–23] or fungal infections such as *Aspergillus* [24] or *Pneumocystis* [25]. It has been proposed that activation of the immune system with increased levels of tumor necrosis factor-*α*, interferon-*γ*, and interleukins IL-2, as well as monocytes, macrophages, natural killer cells, dendritic cells, and B- and T-lymphocytes [26]. Macrophages, NK cells, and T-lymphocytes are activated, and TLR-activated regulatory T-cells are prevented from inhibiting specific cytotoxic T-cells [27]. Prolonged exposure to TLR agonists sensitizes tumor cells to killing by immune mechanisms, especially cytotoxic T-cells. In vitro leukemic cells become more sensitive to cytotoxic T-cells after several days of activation by TRL agonists [28].

BCG stimulates TRL2 and TRL4 via its mycobacterial components, including cell wall skeleton and peptidoglycan. It also stimulates TRL9 via its bacterial DNA [29, 30]. BCG stimulates TRAIL/Apo-2L secretion from neutrophils, as do TRL2 and TRL4 agonists. TRAIL/Apo-2L recruits macrophages and cytotoxic T-cells and may be a mechanism of its anticancer effect [31].

This could be a possible mechanism for the changes seen during BCG treatment, the decrease in clonal myelodysplastic cells permitting more normal hematopoiesis. This decrease in myelodysplastic cells would also explain the change from a hypercellular to hypocellular bone marrow. After finishing treatment, there was no longer TRL agonist stimulation permitting a slow recuperation of the myelodysplastic clone and return to pretreatment levels of hemoglobin and platelet counts. The fact that there was no evidence of tumor relapse supports the hypothesis of an effect of BCG therapy rather than an effect of the bladder cancer. The patient had no evidence of systemic infection or inflammatory disease during the remission period.

A second possibility is the use of blood transfusions; cytotoxic antibodies against leukemic cells have been previously described [32] or transfused allogeneic lymphocytes causing a “graft versus leukemia effect” [33].

However, in cases of spontaneous remission in patients with myelodysplasia, there was not an association with sepsis [10]. In this group of patients, the median time from diagnosis was 18 months with a median duration of transfusional support was 15 months. In the case reported, the patient had been diagnosed seven years previous to the BCG admission, the remission lasted approximately the same time period as BCG treatment. The inference being that the BCG caused the transient remission.

In summary, a patient with transfusion-dependent myelodysplasia was diagnosed with bladder cancer. With transfusional support, the tumor was successfully resected, requiring two operations. During the administration of intravesical BCG therapy, the full blood count normalized and transfusions were not required. We postulate that a TRL agonist effect was produced by the BCG, affecting clonal myelodysplastic stem cells, permitting the normalization of the full blood count. After cessation of BCG therapy, the full blood count returned to pretreatment values and transfusion dependence.
